# Integrative Proteomic Analysis Reveals VMG Oncolytic Virus–Induced Molecular Reprogramming in Pancreatic Ductal Adenocarcinoma

**DOI:** 10.21203/rs.3.rs-9943137/v1

**Published:** 2026-06-22

**Authors:** Sina Aslanabadi, Amirsalar Mansouri, Olivia Hart, Aleksandra Cios, Conner Hartupee, Dicle Yalcin, Garima Sinha, Dorota Wyczechowska, Zetao Cheng, Sudhakar Ammanamanchi, Jovanny Zabaleta, John West, Mitesh Borad, Bolni Marius Nagalo, Jiri Adamec, Omeed Moaven

**Affiliations:** 1 Department of Interdisciplinary Oncology, Louisiana State University (LSU) Health School of Medicine- New Orleans, LA, United States; 2 LSU-LCMC Cancer Center, New Orleans, LA, United States; 3 Marlene and Stewart Greenebaum NCI Comprehensive Cancer Center, University of Maryland School of Medicine, Baltimore, MD, United States; 4 Department of Internal Medicine, Tulane University, New Orleans, LA, United States; 5 Division of Surgical Oncology, Department of Surgery, Louisiana State University (LSU) Health- New Orleans, LA, United States; 6 Division of Hematology/Oncology, Department of Internal Medicine, Mayo Clinic, Phoenix, AZ, United States; 7 Department of Pharmacology and Physiology, University of Maryland, Baltimore, MD, United States

## Abstract

Pancreatic ductal adenocarcinoma (PDAC) is an aggressive malignancy with a 5-year survival rate of 13.3%. This study investigates the treatment efficacy of VMG, a chimeric oncolytic vesiculovirus, against PDAC, focusing on its mechanism and therapeutic potential. VMG is engineered to replace the Vesicular Stomatitis Virus (VSV) glycoprotein (G) gene with the Morreton virus (MorV) glycoprotein, enhancing its safety, efficacy, and immune infiltration compared to wild-type VSV.

We conducted an in-depth proteomic analysis of VMG treatment and evaluated its in vitro and in vivo efficacy. Our findings indicate that VMG treatment not only induces apoptosis and reduces tumor cell viability in PDAC cell lines, but also significantly slows tumor growth and enhances survival in PDAC mouse models.

We then explored whether proteomic analysis could help uncover the mechanistic underpinnings of this efficacy. This approach allowed us to investigate the biological pathways involved in VMG’s action.

To understand these mechanisms, proteomic data were integrated with information from Ingenuity Pathway Analysis (IPA), the Kyoto Encyclopedia of Genes and Genomes (KEGG), and The Cancer Genome Atlas (TCGA). This analysis aimed to identify the key signaling pathways modulated by VMG. By integrating efficacy testing with proteomic and pathway analyses, we sought to clarify how VMG exerts its molecular effects. This approach offers a strong framework for uncovering the biological processes influenced by VMG.

The study reveals that VMG treatment leads to a substantial overlap between virus-induced proteomic changes and prognostic markers found in the TCGA PDAC cohort. Specifically, VMG downregulates proteins associated with unfavorable prognosis, including those involved in altered mitochondrial function, genome DNA synthesis and repair, RNA modification, ribosome biogenesis, intracellular trafficking, cell cycle control, cytoskeletal dynamics, and palmitoylation. This multifaceted oncolytic mechanism disrupts fundamental processes driving PDAC progression, survival, and chemoresistance, providing a robust foundation for identifying novel therapeutic targets and biomarkers.

## Introduction

Pancreatic ductal adenocarcinoma (PDAC) is one of the most aggressive malignancies, with a 5-year overall survival rate of only 13.3% ^[1]^. At diagnosis, 80–85% of PDAC patients have advanced or metastatic disease, rendering their tumors not a candidate for surgical resection; among the minority eligible to undergo surgery, only 20% survive 5 years after the operation ^[2]^.

The current standard of care for PDAC is surgery if possible and systemic treatment with chemotherapy for all patients; however, available regimens provide only modest benefit, with median overall survival remaining below 12 months ^[3]^. Immunotherapy treatments have shown great promise for the treatment of several cancer types, however their efficacy in PDAC is severely limited by its immunosuppressive tumor microenvironment (TME) ^[4]^. There is therefore an urgent need for novel therapeutic approaches to PDAC.

Oncolytic viruses (OV) represent a promising emerging therapeutic option for multiple malignancies including PDAC. OVs can selectively replicate within tumor cells, either through inherent properties or genetic engineering, and exert direct cytolytic effects while simultaneously stimulating the antitumor immune response ^[5]^. ‘In 2015, the first oncolytic virus, T-VEC (a recombinant herpes simplex virus), was approved by the U.S. FDA to treat advanced melanoma ^[6]^. The approval of T-VEC catalyzed widespread interest in exploring oncolytic viruses as potential treatments for a broad range of other malignancies.

Among oncolytic viruses, VSV has shown particular promise for treating multiple cancer types. VSV is an RNA virus that causes disease in livestock; therefore, pre-existing immunity is rare in humans ^[7]^. In normal noncancerous cells, VSV infection is effectively suppressed and eliminated by a type I interferon (IFN) antiviral response ^[7]^. However, the type I IFN response is often downregulated in cancer cells, including those in PDAC tumors, allowing for oncoselectivity of VSV treatment ^[7,8]^. Additionally, VSV has a small and easily manipulable genome, allowing for efficient creation of genomically stable recombinants with reduced neurotropic effects and increased safety ^[9]^. VMG, a chimeric virus created by replacing the VSV glycoprotein (G) gene with the Morreton virus (MorV) gene, has demonstrated improved safety, efficacy, and immune infiltration compared to VSV in sarcoma and PDAC models ^[10,11]^.

In this study, we present an in-depth proteomic analysis of VMG treatment and investigate its in vitro and in vivo efficacy. By integrating our findings with data from Ingenuity Pathway Analysis (IPA), the Kyoto Encyclopedia of Genes and Genomes (KEGG), and The Cancer Genome Atlas (TCGA), we identified key signaling pathways altered by VMG treatment in PDAC cells. These findings provide insight into the molecular mechanism by which VMG inhibits PDAC cell growth and highlight its potential as a therapeutic strategy. Our results provide a mechanistic foundation for the rational development of novel therapeutic strategies targeting PDAC.

## Materials and Methods

### Study Design and Cell Preparation

MiaPaCa2 pancreatic cancer cell lines were procured from ATCC (American Tissue Culture Collection). Cells were cultured in Dulbecco’s Modified Eagle Medium (DMEM) supplemented with 10% Fetal Bovine Serum (Sigma-Aldrich F2442) and grown to 70%−90% confluency in 6-well culture dishes. For the experimental treatment, cells were infected with VMG at a multiplicity of infection (MOI) of 1, a dose pre-optimized in BHK-21 cells to ensure consistent viral activity and provide a standardized reference for infection across different cell lines.

### Protein Extraction and Digestion

For protein extraction, MiaPaCa2 cell samples were treated with an equal volume (in μL) of ABC Buffer (100 mM Ammonium Bicarbonate, pH 8) and eight volumes of 100% Methanol. Samples underwent homogenization using a Bead Ruptor 96 (OMNI International, USA) with six large beads. The resulting suspensions (180 μL) were transferred to new Eppendorf tubes, centrifuged at 14,000×*g* for 5 minutes, and the supernatant was discarded. Pellets were washed twice with cold acetone and dried using a SpeedVac concentrator.

For digestion, protein pellets were solubilized in 20 μL of Denaturation Buffer (25 mM ammonium bicarbonate, pH 8.0; 10 mM TCEP; 5% SDC [sodium deoxycholate]), incubated for 10 minutes at 60°C, and alkylated with 5 μL of freshly prepared Alkylation Buffer (100 mM Iodoacetamide in water). Incubation continued for an additional 60 minutes at room temperature in the dark. Post-alkylation, samples were diluted with 175 μL of dilution buffer (25 mM ammonium bicarbonate, pH 8.0), and 2 μL of Trypsin solution (1μg/μL) were added. Samples were incubated overnight at 37°C. To halt the reaction and remove SDC, 10 μL of 10% TFA was added, and the mixture was incubated for 30 minutes at room temperature before centrifugation at 15,000×*g* (SDC precipitates at low pH). Supernatants were then transferred to new tubes for direct LC-MS analysis.

### LC-MS/MS Analysis

All analyses were carried out using a Bruker nanoElute2 System coupled to a timsTOF fleX 2 mass spectrometer. The mobile phases were composed of Solvent A (0.1% formic acid in 3% ACN) and Solvent B (0.1% formic acid in ACN). The injection volume was 2 μL. Following injection, the peptides were separated on a C-18 reversed-phase PepSep column (0.150 × 250 mm; 1.5 μm particle; Bruker) coupled with the CaptiveSpray ionization source of the mass spectrometer. The flow rate and temperature of the column chamber were set to 800 nL/min and 50°C. Separation of peptides was achieved at the following gradient: T=0 min: 0% B; T=40 min: 26% B; T=40.5 min: 95% B; T=41.5 min: 95% B; T=42 min: 0% B; T=45 min: 0% B (column re-equilibration). Mass spectrometry data was collected in positive, Data Dependent Acquisition (DDA) – PASEF mode under the following conditions: a capillary voltage of 1,500 V; source temperature of 180°C; dry gas flow at 3 L/min; and an acquisition range of 100 – 1,700 m/z. The time settings were as follows: 1/K0 Start: 0.60 Vs/cm^2^; 1/K0 End: 1.60 Vs/cm^2^; Ramp Time: 100 ms; Accumulation Time: 100 ms; Duty Cycle: 100%; and Ramp Rate: 9.42 Hz.

### Data Analysis

Proteomic data were initially processed using MaxQuant ^[13]^, leveraging the UniProt *Homo sapiens* database to identify proteins using label-free quantification (LFQ). LFQ intensities were processed and prepared using an in-house R script. Proteins detected in at least five out of six replicates of samples in any given group were classified as detected proteins for that group. The median gap-filling method addressed potential gaps in protein intensity measurements. Proteins detected in at most two out of six replicates in a group were classified as undetected proteins for the corresponding group. To reduce uncertainty in protein detection levels, proteins that do not fall into either the undetected or detected categories were excluded for a given group. Statistical analyses were conducted using the R limma package (limma_3.58.1) ^[14]^ to identify differentially expressed proteins, with the alpha level set at a p-value of less than 0.05 and absolute log2-transformed fold change (log2FC) > 0.585 (corresponding to FC of 1.5). For downstream analysis, Ingenuity Pathway Analysis (IPA, Qiagen), KEGG pathway analysis, and Gene Ontology (GO) analysis using the DAVID Bioinformatics Resources ^[15,16]^ were used to identify enriched pathways and interpret underlying biological processes.

#### Apoptosis Assay using Red Annexin V Dye with IncuCyte SX5

Apoptosis was quantified using the IncuCyte SX5 Live-Cell Analysis System (Sartorius) with IncuCyte Annexin V Red reagent (Sartorius, Cat No.4642). MiaPaCa2, BXPC-3, Capan-1, and HPAF-II PDAC cell lines were seeded in 96-well clear-bottom plates at a density optimized for each cell line to ensure sub-confluent growth during the experiment. Following cell adherence, cells were treated with VMG-GFP at various MOIs (0.1, 1, and 10), alongside appropriate controls (untreated cells and vehicle-treated cells). Immediately after treatment, IncuCyte Annexin V Red reagent was added to each well at a final concentration of 5% (v/v) according to the manufacturer’s instructions.

The plates were then placed into the IncuCyte SX5 system, maintained at 37°C in a humidified 5% CO2 atmosphere. Images were captured every 2 hours for a total duration of 72 hours, using both phase contrast and red fluorescence channels. Apoptotic cells, labeled with Annexin V Red, were automatically identified and quantified by the IncuCyte software as “red object count per image.” Data were analyzed to assess the kinetics of apoptosis induction in response to VMG-GFP treatment across the different cell lines.

#### Real-time Viral Kinetics with IncuCyte SX5

Cells were infected with VMG-GFP, an oncolytic virus engineered to express Green Fluorescent Protein (GFP), at a predetermined multiplicity of infection (MOI: 1) based on preliminary optimization studies. Immediately following infection, the plates were transferred to the IncuCyte SX5 Live-Cell Analysis System (Sartorius). The IncuCyte system was programmed to capture images every 2 hours for a total duration of 72 hours.

Viral infiltration and replication were monitored in real-time by quantifying two key metrics: phase object confluence and green fluorescence intensity.

Phase object confluence, representing the overall cell density and morphology, was measured using the IncuCyte “Phase Object Count” property. This provided an indirect measure of cell viability and potential cytopathic effects induced by the virus. Viral replication within cells was directly assessed by measuring the “Green Image Mean” property, which quantifies the average GFP fluorescence intensity per image. An increase in green fluorescence intensity indicated successful VMG-GFP infection, followed by viral protein expression and replication. Data from both metrics were collected and analyzed over the 72-hour period to establish the kinetics of VMG-GFP infection and its impact on the ASPC-1, MiaPaCa-2, HS766T, and KPC cell lines.

#### Real-time Viral Kinetics in 3D Spheroids using IncuCyte System

Panc-01 cells were cultured in appropriate growth media supplemented with 10% Fetal Bovine Serum (FBS) and maintained at 37°°C in a humidified 5% CO2 incubator. To generate 3D spheroids, Panc-01 cells were seeded in ultra-low attachment round-bottom 96-well plates (e.g., Corning Spheroid Microplates) at a density optimized for consistent spheroid formation, typically 2,000–5,000 cells per well. The plates were then briefly centrifuged to promote initial aggregation and incubated for 3–5 days to allow spheroid formation and compaction prior to viral infection. Spheroid formation was monitored under a microscope to ensure uniform size and morphology. Panc-01 3D spheroids were infected with VMG-GFP, an oncolytic virus engineered to express Green Fluorescent Protein (GFP), at a predetermined multiplicity of infection (MOI: 1) optimized for 3D culture models.

Following infection, the 96-well plates containing the spheroids were transferred to the IncuCyte SX5 Live-Cell Analysis System (Sartorius). The IncuCyte system was programmed to acquire images at regular intervals, specifically every 2 hours, over a 180-hour period, to comprehensively observe VMG-GFP infiltration and replication within the 3D spheroid.

Real-time viral kinetics was assessed by monitoring the increase in green fluorescence intensity within the spheroids, indicating VMG-GFP infection and subsequent protein expression. The IncuCyte software’s capabilities for analyzing 3D structures were utilized to quantify changes in green signal over time, providing insights into the spatial and temporal dynamics of viral spread and oncolysis within the complex spheroid microenvironment. Visual observation of GFP signal progression allowed for the qualitative assessment of VMG-GFP infiltration into the Panc-01 cells forming the spheroid.

#### In vivo experiments

Two separate animal experiments were performed using C57BL/6 mice (n = 14 per experiment). KPC cells were cultured under standard conditions (DMEM with 10% FBS, 1% penicillin-streptomycin, at 37°C in 5% CO_2_) and prepared at a concentration of 1×10^6^ cells per injection. Mice were subcutaneously injected with 1×10^6^ KPC cells in the flank on Day 0. Tumor growth was monitored regularly, and when tumor volumes reached 50–100 mm^3^ (usually between Days 7 and 10), mice received their first intratumoral injection.

Injected agents included purified VMG-GFP (in vivo grade) with the TCID50 of 1×10^8^ and phosphate-buffered saline (PBS) as a control. TCID50 was determined in BHK-21 cells using the Spearman-Karber method over 7 days, as described by Reed and Muench (1938), for the VMG-GFP in vivo grade virus. Injections were administered twice weekly for 3 weeks, totaling 6 intratumoral injections, with the first on Day 10 and the last on Day 28.

In the initial experiment, mice were sacrificed 24 hours after the sixth intratumoral injection (Day 29) by humane euthanasia. Tumor were excised, fixed in formalin, and processed into formalin-fixed paraffin-embedded (FFPE) sections for subsequent histological and molecular analyses. A second experiment followed the same treatment protocol but was evaluated as a survival study. Mice were monitored after the final injection for survival endpoints and euthanized upon reaching humane criteria or natural death.

All animal procedures were approved by the appropriate institutional animal care and use committee (IACUC Protocol #4908) and were performed in accordance with ethical guidelines.

#### Tumor models and treatment for flow cytometry:

##### Subcutaneous KPC tumor model

KPC Y pancreatic cancer cells were injected subcutaneously into the right flanks of female C57BL/6J mice (Jackson Laboratory, strain #000664; n = 8 per group). Tumor growth was monitored until average tumor volumes reached approximately 80–120 mm^3^. Mice were then treated via intratumoral injection with 50 μL of either PBS (vehicle control) or VMG GFP at a dose of 1 × 10^7^ TCID_50_. Treatments were administered twice weekly for two consecutive weeks. Tumors were harvested at the experimental endpoint for downstream immunological analyses.

##### Orthotopic KPC tumor model

Exponentially growing, bioluminescent KPC cells (EUP012 FP; Kerafast), derived from a genetically engineered mouse model harboring canonical pancreatic cancer mutations and exhibiting extensive stromal deposition and metastatic potential, were prepared for orthotopic implantation. Cells were harvested by trypsinization, washed, and resuspended in basement membrane–supplemented medium. Male C57BL/6J mice (6–8 weeks of age; Jackson Laboratory, strain #000664) received intrapancreatic injections of 5 × 10^5^ cells in a total volume of 30 μL.

Tumor establishment was monitored by palpation and/or bioluminescence imaging (BLI). Once tumors reached approximately 3 mm in diameter, mice were randomized into treatment groups (n = 10–12 per group) and administered four intraperitoneal injections of PBS or VMG GFP (1 × 10^7^ TCID_50_ in 100 μL) according to the treatment schedule. Tumors were collected two days following the final injection. Tumor progression was monitored longitudinally using BLI, and animals were assessed regularly for weight and clinical signs in accordance with institutional humane endpoint criteria.

All animal procedures were approved by the University of Arkansas for Medical Sciences Institutional Animal Care and Use Committee (IACUC) and conducted in compliance with institutional ethical guidelines.

##### Tumor dissociation and flow cytometry

Tumor infiltrating leukocytes were isolated by enzymatic and mechanical dissociation using the gentleMACS Dissociator (Miltenyi Biotec) in combination with the Mouse Tumor Dissociation Kit (Miltenyi Biotec), according to the manufacturer’s protocol. Resulting cell suspensions were filtered through a 30 μm mesh strainer, washed with PBS containing 1% fetal calf serum, and centrifuged at 500 × g for 5 minutes at 25°C.

Cell counts and viability were assessed using trypan blue exclusion on a Countess 3 Automated Cell Counter (Thermo Fisher Scientific), with typical viabilities of approximately 70%. Cells were stained with fluorochrome conjugated antibodies at optimized concentrations (0.5 μL viability dye and 1 μL of each antibody per 10^6^ cells). The full antibody panel is listed in Table S1.

Following staining, cells were washed and incubated with True Stain Monocyte Blocker (BioLegend) to minimize nonspecific binding. Samples were fixed and acquired on a Cytek Northern Lights flow cytometer at the UAMS Flow Cytometry Core Facility. Flow cytometry data were analyzed using FlowJo software (version 10.10; BD Biosciences).

##### Statistical analysis

Statistical analyses were performed using GraphPad Prism software (version 10.2.3). Data are presented as mean ± standard deviation (SD). Comparisons between groups were conducted using one way ANOVA followed by Dunnett’s multiple comparisons test. Statistical significance was defined as p < 0.05.

##### Gating strategy and identification of tumor infiltrating immune populations

Single cell suspensions derived from tumor tissues were analyzed using a 16 color flow cytometry panel (Table 1) designed to enable comprehensive immunophenotyping of tumor infiltrating leukocytes. Cell size and granularity were first used to exclude debris and doublets, followed by live/dead discrimination to identify viable cells. Immune cells were distinguished from tumor cells based on CD45 expression.

T lymphocytes were identified as CD45^+^CD3^+^ cells and further subdivided into CD4^+^ helper T cells (CD4^+^CD8^−^), CD8^+^ cytotoxic T cells (CD4^−^CD8^+^), and CD4^+^CD8^+^ double positive T cells. Within T cell subsets, expression of CD44, CD69, PD 1, ICOS, and Ki 67 was used to assess activation, differentiation, and proliferative status.

From the CD45^+^CD3^−^ compartment, myeloid populations were identified based on CD11b expression. Myeloid derived suppressor cell subsets were defined by Ly6C and Ly6G expression. Macrophages were identified as CD11b^+^F4/80^+^ cells and stratified into M1 (I A/I E^+^CD206^−^) and M2 (I A/I E^+^CD206^+^) subsets. Conventional dendritic cells were identified based on CD11c expression, while NK cells were defined by CD335 expression.

#### Cell Tox/Viability

Three different pancreatic cancer cell lines — MiaPaCa2, HS766T, and ASPC-1 — were used. VMG-GFP virus stock was prepared for infection at multiplicities of infection (MOI) of 1, 0.1, and 0.01. Cell viability was assessed using the MTS Cell Titer Glo assay kit, following the manufacturer’s protocol. Standard cell culture materials (culture media for MiaPaCa-2 and HS766T: DMEM with 10% FBS; ASPC-1: RPMI-1640 with 10% FBS), including 96-well plates and media optimized for each cell line, were used.

Cells were seeded in 96-well plates and cultured under optimum conditions until they reached approximately 70% confluency. Following this, the cells were infected with VMG-GFP at MOI values of 1, 0.1, and 0.01. Control wells were left uninfected. After 72 hours post-infection, cell viability was measured using the MTS Cell Titer Glo assay according to the manufacturer’s instructions. Each condition was conducted with six replicates to ensure statistical robustness. Viability results were normalized to the uninfected control cells, which were assigned 100%.

## Results

### VMG experiments

**Figure 2.**
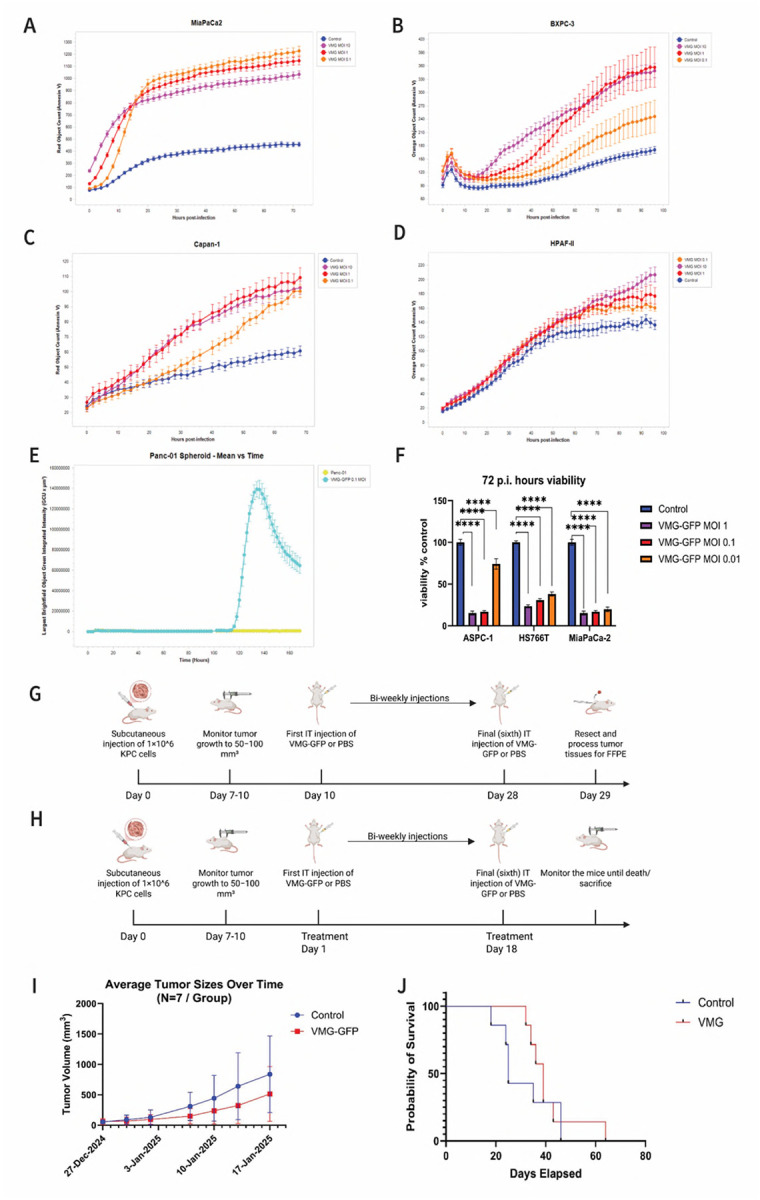
Treatment of PDAC cells with VMG-GFP oncolytic virus induces apoptosis, reduces tumor cell viability, and suppresses tumor progression in preclinical models. A-D) Annexin V Apoptosis assays in PDAC cell lines following VMG-GFP treatment. Annexin V-positive cell count (red/orange object count per image) was measured over 72 hours post-infection with VMG-GFP at MOI 0.1, 1, and 10 in A) MiaPaCa2, B) BXPC-3, C) Capan-1, and D) HPAF-II cells. E) Real-time viral kinetics of VMG-GFP infiltration into a Panc-01 3D spheroid model, monitored using the IncuCyte live-cell imaging system over 180 hours post-infection. F) Cell viability of PDAC cell lines (ASPC-1, HS766T, and MiaPaCa2) was measured 72 hours after treatment with VMG-GFP at MOI 0.01, 0.1, and 1. G) Schematic of the experimental design for in vivo tumor volume assessment following VMG-GFP treatment. H) Schematic of the in vivo survival study timeline following VMG-GFP treatment. I) Average tumor volume in PDAC mice beginning on Day 1 of VMG-GFP treatment (n = 7 per group). I) Kaplan-Meier survival analysis of PDAC mice following the final (sixth) intratumoral injection of VMG-GFP (n=7).

We first evaluated the efficacy of VMG treatment both in vitro and in vivo. Cell toxicity results indicated a significant decrease in cell viability across all three cell lines upon infection with VMG-GFP at all MOI values compared to the control (p < 0.0001)(Figure 2-F). ASPC-1 cells showed the greatest reduction in viability, particularly at MOIs of 1 and 0.1, with viability dropping to approximately 15–20% of control levels (Figure 2-F). HS766T cells showed moderate susceptibility, with viability ranging from 20% to 35% across MOIs. MiaPaCa-2 cells exhibited similar trends, with viability reduced to roughly 20–30%. Despite variation across cell lines and MOIs, all infected groups showed statistically significant reductions in viability compared to controls. The results confirm that VMG-GFP, even at the lowest MOI tested, effectively reduces the viability of these pancreatic cancer cell lines after 72 hours (Figure 2F). Analysis of PDAC cell lines MiaPaCa2, BXPC-3, Capan-1, and HPAF-II with Annexin V showed that treatment with VMG-GFP at various MOIs induced apoptosis in MiaPaCa2, BXPC-3, and Capan-1 cell lines (Figure 2A–D). IncuCyte viral kinetics analysis of VMG infection and replication in a Panc-01 three-dimensional spheroid model demonstrated that VMG can effectively and rapidly infect PDAC cells (Figure 2E). Finally, in vivo studies assessing tumor growth and survival indicated that VMG treatment not only slows PDAC tumor growth but also enhances survival in PDAC mice (Figure 2I–J).

### VMG GFP alters immune composition in subcutaneous KPC tumors

**Figure 3.**
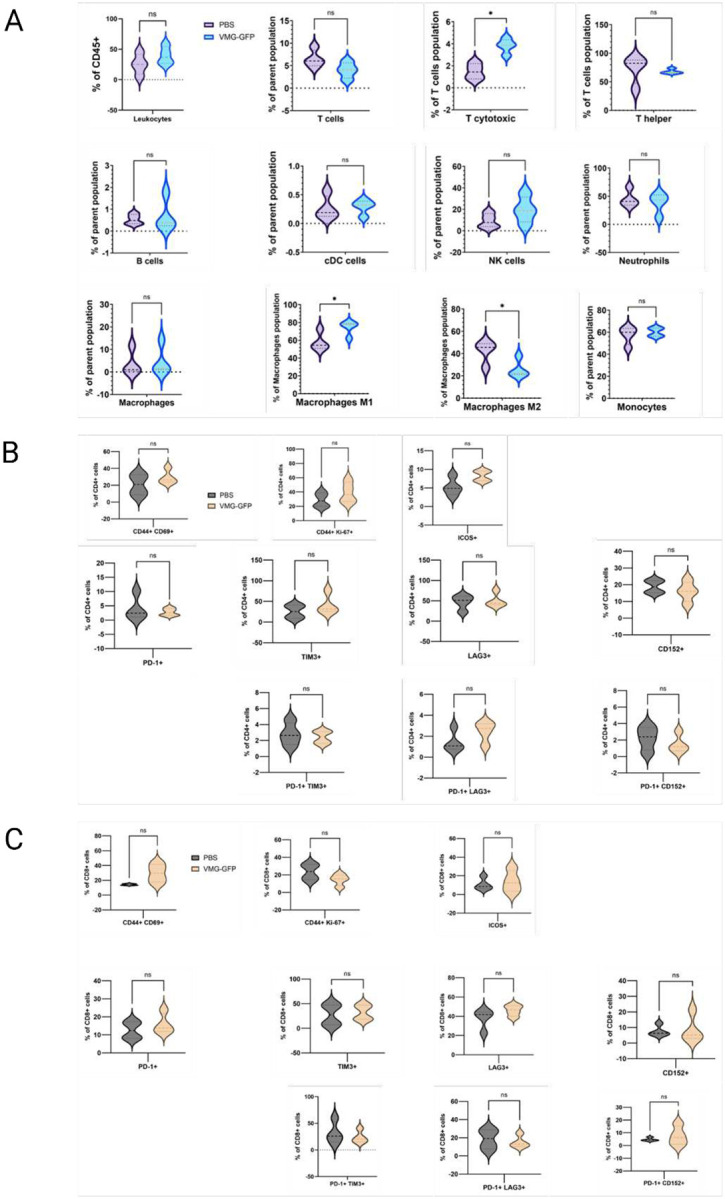
VMG GFP alters immune composition in subcutaneous KPC tumors. A)Immune cell composition in subcute KPC tumors following VMG-GFP treatment. Violin plots show the relative frequencies of major tumor-infiltrating leukocyte subpopulations, including T cells, B cells, cytotoxic and helper T cells, dendritic cells, NK cells, neutrophils, macrophage subsets, and monocytes, in PBS- and VMG-GFP–treated mice. Each plot represents the distribution of individual tumors; statistical significance is indicated. B)Functional and inhibitory marker expression on tumor-infiltrating CD4^+^ T cells. Violin plots depict the frequency of activated, proliferating, and inhibitory receptor–expressing CD4^+^ T cells within orthotopic KPC tumors from PBS- and VMG-GFP–treated mice, including ICOS, Ki-67, PD-1, TIM-3, LAG-3, CD152, and selected co-expression profiles. C)Functional and inhibitory marker expression on tumor-infiltrating CD8^+^ T cells. Violin plots show the distribution of activation, proliferation, and inhibitory/exhaustion marker expression on CD8^+^ T cells isolated from orthotopic KPC tumors following PBS or VMG-GFP treatment, including ICOS, Ki-67, PD-1, TIM-3, LAG-3, CD152, and combinatorial marker expression.

Tumor infiltrating immune cells were analyzed in subcutaneous KPC tumors following treatment with PBS or VMG GFP. VMG GFP treatment did not significantly affect overall immune infiltration, as frequencies of total leukocytes (CD45^+^CD3^−^), total T cells, and B cells were comparable between treatment groups. In contrast, VMG GFP significantly increased the proportion of cytotoxic T cells (CD8^+^ T cells as a fraction of total T cells), whereas the frequency of helper T cells (CD4^+^) remained unchanged (Figure 3A).

Phenotypic profiling of tumor infiltrating CD4^+^ and CD8^+^ T cells revealed no significant differences between groups in markers of activation (CD44^+^CD69^+^), proliferation (CD44^+^Ki 67^+^), or costimulatory signaling (ICOS). Expression of inhibitory and exhaustion associated receptors, including PD 1, TIM 3, LAG 3, and CD152, as well as their co expression patterns, was also unchanged in both T cell subsets (Figure 3B).

Analysis of the innate and myeloid compartments demonstrated preserved frequencies of conventional dendritic cells, NK cells, neutrophils, monocytes, and total macrophages in VMG GFP–treated tumors. Notably, macrophage subset analysis revealed a significant increase in M1 macrophages, a concomitant decrease in M2 macrophages, and a corresponding increase in the M1/M2 ratio, indicating a shift toward a pro inflammatory macrophage phenotype in subcutaneous tumors (Figure 3C).

### VMG GFP selectively modulates adaptive immunity in orthotopic KPC tumors

**Figure 4.**
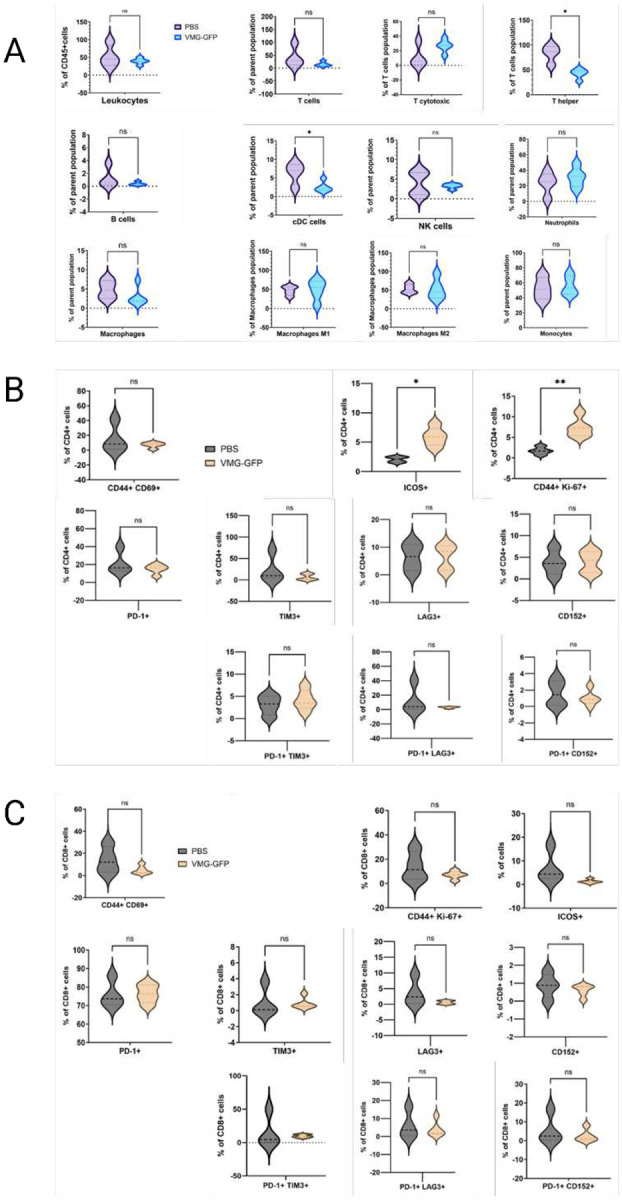
VMG GFP selectively modulates adaptive immunity in orthotopic KPC tumors. A)Immune cell composition in orthotopic KPC tumors following VMG-GFP treatment. Violin plots show the relative frequencies of major tumor-infiltrating leukocyte subpopulations, including T cells, B cells, cytotoxic and helper T cells, dendritic cells, NK cells, neutrophils, macrophage subsets, and monocytes, in PBS- and VMG-GFP–treated mice. Each plot represents the distribution of individual tumors; statistical significance is indicated. B)Functional and inhibitory marker expression on tumor-infiltrating CD4^+^ T cells. Violin plots depict the frequency of activated, proliferating, and inhibitory receptor–expressing CD4^+^ T cells within orthotopic KPC tumors from PBS- and VMG-GFP–treated mice, including ICOS, Ki-67, PD-1, TIM-3, LAG-3, CD152, and selected co-expression profiles. C)Functional and inhibitory marker expression on tumor-infiltrating CD8^+^ T cells. Violin plots show the distribution of activation, proliferation, and inhibitory/exhaustion marker expression on CD8^+^ T cells isolated from orthotopic KPC tumors following PBS or VMG-GFP treatment, including ICOS, Ki-67, PD-1, TIM-3, LAG-3, CD152, and combinatorial marker expression.

In orthotopic KPC tumors, VMG GFP treatment did not significantly alter the overall abundance of tumor infiltrating leukocytes, total T cells, or B cells relative to PBS controls. The proportion of cytotoxic T cells within the T cell compartment was unchanged, whereas the frequency of helper T cells was significantly reduced(Figure 4A).

Despite this reduction, CD4^+^ T cell phenotyping revealed selective functional modulation, characterized by significantly increased ICOS expression and enhanced proliferation (CD44^+^Ki 67^+^). Expression of early activation markers (CD44^+^CD69^+^) and inhibitory or exhaustion associated receptors (PD 1, TIM 3, LAG 3, CD152), including their co expression, was not significantly altered. CD8^+^ T cells similarly exhibited no significant changes in activation, proliferation, ICOS expression, or inhibitory receptor expression (Figure 4B).

Innate immune profiling demonstrated preserved frequencies of NK cells, neutrophils, monocytes, total macrophages, and macrophage M1/M2 polarization following VMG GFP treatment. In contrast, the frequency of intratumoral conventional dendritic cells was significantly reduced. Importantly, this reduction occurred alongside intact innate immune composition and enhanced functional activation of CD4^+^ T cells (Figure 4C).

### Differential Protein Expression and Functional Enrichment Analysis

Proteomic analysis of cultured cell samples detected a total of 3,935 proteins in the VMG-treated group and 3,627 proteins in the control group. Of detected proteins, 3,452 (87.7% of VMG-treated and 95.2% of control) were detected in both the VMG-treated and control groups. In comparison, 483 proteins (12.3%) were detected only in the VMG-treated group, and 175 proteins (4.8%) were detected only in the control group (Figure 2A). Among the significantly dysregulated proteins, 123 were upregulated, and 125 were downregulated (Figure 2B). Table 1 presents the top 5 GO-enriched terms for the 51 commonly detected proteins that are significantly dysregulated across the Cellular Component (CC), Biological Process (BP), and Molecular Function (MF) domains. The complete GO enrichment analysis results are provided in Table S2.

To elucidate the biological significance of VMG-treatment-induced proteomic alterations, we performed pathway enrichment analyses using the Kyoto Encyclopedia of Genes and Genomes (KEGG) and Ingenuity Pathway Analysis (IPA). KEGG results (Table 2) revealed significant enrichment of pathways involved in DNA replication and repair, oxidative phosphorylation, metabolic reprogramming, and cell-cycle regulation, indicating that VMG disrupts key processes supporting PDAC growth and survival. Complementary IPA canonical pathway analysis (Table 3) corroborated these findings and further revealed VMG-induced perturbation of cell-cycle progression, DNA damage response, and transcriptional regulation, including activation of the mitotic G1/S transition, nucleotide excision repair, and RNA polymerase II-dependent transcription pathways (Table 3). Additionally, enrichment of protein ubiquitination, peroxisomal lipid metabolism, and class I MHC-mediated antigen processing suggests broader remodeling of proteostasis and immune recognition mechanisms in VMG-treated PDAC cells.

### TCGA Analysis

By cross-referencing the defined significantly dysregulated proteins of our experiment with the TCGA metadata study, we investigate the overlap of significantly regulated proteins after the infection with VMG with the potential and validated prognostic genes from the metadata study in Human Atlas Proteome, which used data from TCGA, where transcriptomics data of 176 patients in total ^[12]^. Table 4 presents the overlap between significantly dysregulated proteins and the TCGA pancreatic adenocarcinoma metadata.

## Discussion

The current study employs an integrative proteomic analysis of VMG-infected MiaPaCa-2 cells, a representative model of pancreatic ductal adenocarcinoma (PDAC), to reveal a substantial overlap between virus-induced proteomic changes and prognostic markers identified from The Cancer Genome Atlas (TCGA) PDAC cohort ^[17–20]^. This concordance between experimental cellular models and large-scale human patient data provides a robust foundation for identifying novel therapeutic targets and biomarkers for this aggressive malignancy. Our findings indicate that VMG infection drives a molecular shift characterized by the downregulation of 21 proteins previously associated with unfavorable prognosis in PDAC and the upregulation of 3 proteins linked to favorable prognosis, collectively pointing towards a therapeutically beneficial outcome. This comprehensive proteomic reprogramming underscores VMG’s potential as a potent oncolytic agent that can modulate key cellular pathways critical to PDAC progression, survival, and drug resistance. The proteomic signature identified following VMG treatment could inform patient selection criteria for future clinical trials, prioritizing individuals whose tumors exhibit high expression of unfavorable prognostic markers downregulated by VMG (e.g., ATAD2, RRM2) or low expression of favorable markers upregulated by VMG (e.g., TOM1L2), akin to biomarker-driven approaches in, thereby optimizing clinical trial design and patient outcomes.

### VMG-GFP Modulates the Tumor Immune Microenvironment in a Model-Dependent Manner

To complement the proteomic and in vitro findings, we investigated the in vivo immunological consequences of VMG-GFP treatment using comprehensive flow cytometric immunophenotyping of tumor-infiltrating leukocytes in two clinically relevant syngeneic KPC pancreatic cancer models. Our findings reveal that VMG-GFP exerts distinct, context-dependent immunomodulatory effects on both the innate and adaptive immune compartments, consistent with the emerging paradigm of oncolytic viruses as multifaceted immunotherapeutic agents capable of remodeling the tumor immune microenvironment (TIME) ^[21]^.

### VMG-GFP Promotes Cytotoxic T Cell Expansion and Pro-Inflammatory Macrophage Polarization in Subcutaneous KPC Tumors

In subcutaneous KPC tumors, VMG-GFP treatment produced a selective and significant increase in the proportion of CD8^+^ cytotoxic T cells within the tumor-infiltrating T cell compartment, without altering the overall frequencies of total leukocytes, total T cells, or B cells. This pattern is consistent with findings from multiple oncolytic virotherapy studies demonstrating that VSV-based and other oncolytic platforms preferentially recruit and expand CD8^+^ T cells within the tumor microenvironment without causing broad, nonspecific immune infiltration ^[22,23]^. CD8^+^ cytotoxic T cells are the primary effectors of antitumor immunity, capable of directly lysing tumor cells through the release of perforin and granzymes and through Fas-FasL interactions, making their selective expansion by VMG-GFP a particularly favorable immunological outcome ^[24]^. Importantly, the CD8^+^ T cells in VMG-GFP-treated subcutaneous tumors did not exhibit upregulation of canonical exhaustion or inhibitory receptors—including PD-1, TIM-3, LAG-3, and CD152—suggesting that the expanded CD8^+^ population retains functional competence and has not been rendered dysfunctional by the immunosuppressive tumor microenvironment ^[25]^. This observation is of particular translational relevance, as functionally intact, non-exhausted cytotoxic T cells are more likely to mediate durable antitumor responses and to synergize with immune checkpoint blockade therapies ^[26,27]^.

In parallel with the adaptive immune changes, VMG-GFP treatment significantly reprogrammed the intratumoral macrophage pool in subcutaneous tumors, resulting in a marked increase in M1 (IA/IE^+^CD206^−^) macrophages, a concomitant decrease in M2 (IA/IE^+^CD206^+^) macrophages, and an elevated M1/M2 ratio. Tumor-associated macrophages (TAMs) are well established as key determinants of the immunosuppressive tumor microenvironment in PDAC, with the M2-polarized phenotype associated with tumor promotion, angiogenesis, immune evasion, and resistance to therapy ^[28]^. The VMG-GFP-induced shift toward M1 polarization reflects a reprogramming of the TIME toward a pro-inflammatory, antitumorigenic state. This finding is consistent with previous reports demonstrating that VSV and VSV-derivative oncolytic viruses preferentially infect and kill M2 macrophages while inducing M1 repolarization through the type I interferon anti-viral response pathway ^[29]^. Similarly, in a syngeneic KPC model, intratumoral injection of an armed VSV (VSV-S) preferentially eliminated M2-like macrophages over those with an M1 phenotype, resulting in reversion of immunosuppressive conditions that, in combination with anti-PD-1 therapy, achieved long-term survival in a significant proportion of treated animals ^[30]^. The current findings with VMG-GFP extend this observation to a chimeric vesiculovirus platform, suggesting that the M2→M1 repolarization capacity is a conserved property of VSV-based oncolytic viruses, potentially reflecting the intrinsic interferon-inducing capacity of the viral glycoprotein and the selective vulnerability of immunosuppressive M2 macrophages to VSV family members ^[31]^. M1 macrophages contribute to antitumor immunity through the secretion of pro-inflammatory cytokines including TNF-α, IL-12, and IL-6, the enhancement of antigen presentation via MHC-II upregulation, and the direct cytotoxic killing of tumor cells, collectively amplifying the antitumoral T cell response initiated by VMG-GFP infection ^[32,33]^.

### VMG-GFP Selectively Activates CD4^+^ Helper T Cells Without Inducing Exhaustion in Orthotopic KPC Tumors

The orthotopic model, which more faithfully recapitulates the complex stromal and immunosuppressive microenvironment of human PDAC, yielded a distinct immunological profile compared to the subcutaneous setting. VMG-GFP treatment did not significantly alter total leukocyte, T cell, or B cell frequencies in orthotopic tumors, and the proportion of CD8_+_ T cells within the T cell compartment was unchanged. The frequency of CD4^+^ helper T cells was significantly reduced; however, phenotypic profiling revealed that the remaining CD4^+^ T cell population underwent meaningful functional reprogramming, characterized by a significant increase in ICOS expression and enhanced proliferation as measured by Ki-67 positivity. ICOS (Inducible T-cell Co-Stimulator) is a CD28-family co-stimulatory receptor that plays a pivotal role in T helper cell differentiation, survival, and effector function, and its expression is associated with activated, recently antigen-stimulated T cells with enhanced cytokine production capacity ^[34]^. The upregulation of ICOS on intratumoral CD4^+^ T cells following VMG-GFP treatment suggests that despite a numerical reduction in the CD4^+^ compartment, VMG-GFP promotes the selective retention and functional activation of antigen-experienced, ICOS-expressing helper T cells in the orthotopic setting. This finding aligns with evidence that oncolytic viruses can upregulate T cell co-stimulatory receptors, including ICOS, as part of a broader remodeling of the intratumoral immune milieu ^[34,35]^. Importantly, CD4^+^ T cell function in the context of antitumor immunity encompasses not only classical T helper roles—such as licensing of dendritic cells and provision of help to CD8^+^ T cells—but also direct cytolytic activity and the orchestration of macrophage activation, making their functional activation by VMG-GFP a potentially important mechanistic contributor to its therapeutic effects ^[36]^.

Notably, neither the CD4^+^ nor the CD8^+^ T cell populations in orthotopic tumors exhibited significant upregulation of inhibitory or exhaustion-associated receptors, including PD-1, TIM-3, LAG-3, and CD152, either individually or in co-expression patterns. The absence of exhaustion marker upregulation in the context of VMG-GFP treatment is a clinically favorable finding, as immune exhaustion represents a principal mechanism of T cell dysfunction in the PDAC TIME and a major barrier to effective immunotherapy. The preservation of a non-exhausted T cell state suggests that VMG-GFP does not exacerbate the immunosuppressive signals that typically drive T cell dysfunction in this disease context, and may therefore be compatible with or synergistic with immune checkpoint blockade strategies targeting PD-1/PD-L1 or other inhibitory axes.

### Differential Regulation of Conventional Dendritic Cells in the Orthotopic Model

A notable finding in the orthotopic model was the significant reduction in intratumoral conventional dendritic cell (cDC) frequency following VMG-GFP treatment. Conventional dendritic cells are essential orchestrators of adaptive antitumor immunity, responsible for tumor antigen uptake, processing, and cross-presentation to naïve and memory T cells, and their abundance within the TIME is generally associated with favorable prognosis across multiple cancer types ^[37]^. The VMG-GFP-associated reduction in cDC frequency in the orthotopic setting may reflect active infection of intratumoral DCs by the oncolytic virus, a phenomenon previously described for wild-type VSV, which has been shown to directly infect tumor-associated dendritic cells, impairing their viability and migratory capacity to draining lymph nodes and thereby interfering with tumor antigen presentation ^[38]^. This interpretation is consistent with the observation that, despite reduced cDC frequencies, CD4^+^ T cells in the orthotopic tumors exhibited increased ICOS expression and proliferative activity, suggesting that antigen presentation and T cell priming proceeded through alternative antigen-presenting pathways or that the residual, virus-activated DCs were sufficient to maintain T cell stimulation. Alternatively, the reduction in conventional DCs may partly reflect their egress from the tumor following cytokine-mediated activation, a phenomenon observed in several oncolytic virus models where treatment with inflammatory stimuli promotes DC maturation and migration to tumor-draining lymph nodes to prime systemic antitumor responses ^[39]^. Future studies employing longitudinal sampling of tumor-draining lymph nodes and functional assessments of DC maturation status will be essential to resolve these possibilities.

### Model-Dependent Immune Responses and Implications for VMG Therapeutic Development

The contrasting immune profiles observed in subcutaneous versus orthotopic KPC tumor models—namely, CD8^+^ T cell expansion and M1/M2 macrophage repolarization in the former versus selective CD4^+^ T cell functional activation and cDC reduction in the latter—reflect the well-established influence of the tumor microenvironment architecture on the immunological response to oncolytic virus therapy ^[40]^. The orthotopic PDAC model employed in this study was derived from a genetically engineered mouse model harboring canonical KPC mutations (KrasG12D and Trp53R172H) and exhibiting extensive stromal deposition and metastatic potential, features that closely mimic human PDAC pathophysiology and that are known to impose significant barriers to immune infiltration and effector function. The denser extracellular matrix and higher interstitial fluid pressure in orthotopic PDAC tumors may limit viral spread and the magnitude of direct oncolysis, thereby reducing the immunogenic cell death signals necessary to drive robust CD8^+^ T cell expansion and innate immune repolarization, as observed in the more permissive subcutaneous model ^[41]^. Nonetheless, the selective functional activation of CD4^+^ T cells in the orthotopic setting suggests that VMG-GFP retains meaningful immunomodulatory capacity even within this more complex and restrictive microenvironment. Collectively, these observations reinforce the concept that the immunological consequences of oncolytic virotherapy are profoundly shaped by the tumor model context, and underscore the importance of evaluating oncolytic candidates in multiple preclinical models—including orthotopic systems that recapitulate the human disease microenvironment—before translational development ^[39]^.

From a therapeutic strategy perspective, the present findings suggest multiple avenues for combination-based enhancement of VMG-GFP efficacy. The non-exhausted state of tumor-infiltrating T cells in both models, combined with the observed M1 macrophage enrichment in subcutaneous tumors and ICOS upregulation in orthotopic tumors, provides a favorable immunological context for the co-administration of immune checkpoint inhibitors targeting PD-1, PD-L1, CTLA-4, or LAG-3, which have previously demonstrated synergistic efficacy with VSV-based oncolytic platforms in multiple preclinical cancer models. Furthermore, the class I MHC-mediated antigen processing and presentation pathway identified as significantly enriched in our proteomic analysis is mechanistically consistent with the CD8^+^ T cell expansion observed in the subcutaneous model, as enhanced antigen presentation efficiency would be expected to amplify tumor-specific cytotoxic responses generated by VMG-GFP-mediated oncolysis and immunogenic cell death ^[42]^. The favorable convergence of proteomic pathway modulation—specifically the enrichment of MHC class I antigen processing, protein ubiquitination, and immune cell activation pathways—with the observed immunophenotypic changes strengthens the biological coherence of VMG-GFP’s dual oncolytic and immunomodulatory mechanism of action and supports its further development as a precision immunotherapeutic agent for PDAC.

### Downregulated Proteins: Vulnerabilities for Therapeutic Intervention

The 21 proteins found to be downregulated in VMG-infected MiaPaCa-2 cells (MRPL3 (P09001), RRM2 (P31350), ATP2C1 (P98194), Q5F1R6, Q69YN4, etc.) consistently correlate with worse clinical outcomes in the TCGA PDAC cohort, emphasizing their roles as drivers of cancer progression. Their reduction after VMG infection suggests that targeting their activity could offer therapeutic benefits for PDAC patients ^[43–45]^. The main mechanisms are outlined below.

### Epigenetic and Chromatin Remodeling

Several downregulated proteins are involved in epigenetic regulation and chromatin dynamics, critical processes frequently hijacked in cancer.

#### ATAD2

(ATPase family AAA domain-containing protein 2, Q6PL18) is an epigenetic regulator with AAA+ ATPase and bromodomain functions, acting as a transcriptional coactivator for oncogenic transcription factors like MYC. Its overexpression is linked to enhanced proliferation, invasion, and chemoresistance in various cancers, including prostate, breast, colorectal, and hepatocellular carcinoma, often indicating a poor prognosis. The downregulation of ATAD2 by VMG suggests a disruption of these tumor-promoting epigenetic programs, leading to cell cycle arrest, reduced cell proliferation, and diminished migration and epithelial-mesenchymal transition (EMT). Targeting ATAD2’s bromodomain or its ATPase activity is considered a promising but challenging therapeutic strategy^[46–54]^.

#### MORF4L1

(Mortality factor 4-like protein 1, Q9UBU8) is an epigenetic modulator involved in histone acetyltransferase activity and the recruitment of DNA repair factors. It generally functions as a tumor suppressor by inhibiting cell proliferation and migration in several cancers, such as nasopharyngeal carcinoma and hepatocellular carcinoma. It’s observed downregulation in VMG-infected cells is a complex finding, potentially reflecting intricate epigenetic remodeling events during oncolysis that warrant further investigation to clarify its precise role in the context of viral therapy.

#### LRIF1

(Ligand-dependent nuclear receptor-interacting factor 1, Q5T3J3) functions as a transcriptional corepressor and is crucial for accurate chromosome segregation during mitosis through its interaction with heterochromatin protein one alpha (HP1α). It also represses retinoic acid receptor alpha (RARα)-mediated transcriptional activation, which is significant given that RARα stimulation can inhibit PDAC cell growth. The VMG-induced downregulation of LRIF1 may contribute to mitotic defects and inhibition of tumor proliferation by compromising chromosomal stability ^[55–59]^.

#### CEBPZ

(CCAAT/enhancer-binding protein zeta, Q03701) is a transcription factor that stimulates the heat shock protein 70 (HSP70) promoter and is involved in ribosomal RNA (rRNA) processing. It exhibits context-dependent roles in tumorigenesis, acting as either a promoter or suppressor depending on the cancer type and cellular environment. Its downregulation by VMG may lead to altered transcriptional control and reduced tumor cell viability, potentially through impaired rRNA processing and protein synthesis, which is crucial for cancer cell growth ^[60–63]^.

### Mitochondrial Function and Metabolism

Disruption of mitochondrial function represents a critical avenue for VMG’s oncolytic effects.

#### TMEM70

(Transmembrane protein 70, mitochondrial, Q9BUB7) is indispensable for the biogenesis of mitochondrial ATP synthase, the enzyme complex responsible for ATP production via oxidative phosphorylation. Mutations in TMEM70 cause severe mitochondrial diseases characterized by ATP synthase deficiency. ^[64]^. In cancer, its downregulation can lead to metabolic reprogramming, including a shift towards glycolysis (the Warburg effect), which is often associated with tumor progression and drug resistance in PDAC. VMG-induced downregulation of TMEM70 suggests a mechanism by which tumor cell metabolism and survival can be impaired by disrupting mitochondrial energy provision. ^[65,66]^.

#### MRPL3

(Mitochondrial Ribosomal Protein L3) is essential for mitochondrial protein production and energy metabolism, pathways often disrupted in cancer to support quick growth. High MRPL3 levels have been linked to increased tumor metabolism and poor prognosis across various cancers ^[67,68]^. Overexpression of MRPL3 is linked to increased tumor metabolic activity and worse prognoses in cancers such as hepatocellular carcinoma and glioma ^[68,69]^. VMG-mediated downregulation of MRPL3 likely cripples mitochondrial protein production, impeding the cancer cells’ ability to meet their high energy demands and contributing to overall tumor growth inhibition. Targeting mitochondrial function continues to be a promising approach, with ongoing studies into modulators that might indirectly influence MRPL3 activity and make cancer cells more sensitive to treatment ^[70–72]^.

#### SQOR

(Sulfide: Quinone Oxidoreductase) plays a role in mitochondrial sulfide processing, thereby affecting redox balance and cell death. Its dysregulation can alter metabolic processes and promote tumor growth, indicating that inhibiting SQOR may interfere with vital survival pathways in cancer cells ^[73–76]^.

### DNA Synthesis, Repair, and Cell Cycle Control

VMG also targets proteins crucial for maintaining genomic integrity and controlling cell proliferation.

#### RRM2

(Ribonucleotide Reductase M2) is a key enzyme for DNA synthesis and repair, making it critical for rapidly dividing cancer cells. Overexpression of RRM2 contributes to resistance to chemotherapy, especially gemcitabine in PDAC, and is associated with poorer outcomes ^[77–80]^. Using small molecule inhibitors like GW8510 against RRM2 has shown promising results combined with gemcitabine in preclinical studies, enhancing drug sensitivity and reducing tumor growth ^[79,81]^.

#### MIS18A

(Q9NYP9) is a kinetochore protein essential for proper centromere assembly and accurate chromosome segregation during cell division ^[82]^. High levels of MIS18A are associated with increased proliferation and poor prognosis. VMG-induced suppression of MIS18A likely leads to mitotic errors and chromosomal instability, ultimately causing cell cycle arrest and apoptotic cell death in cancer cells.

#### NDC1

(Nuclear Division Cycle 1, Q9BTX1) is an integral membrane protein vital for the assembly of nuclear pores, which regulate nucleocytoplasmic transport. Its elevation in various cancers, including PDAC, promotes cell growth and dissemination, potentially through pathways like PI3K/AKT ^[83]^. VMG’s reduction of NDC1 expression could hinder the critical transport of macromolecules between the nucleus and cytoplasm, thereby slowing cancer cell proliferation and contributing to tumor suppression.

#### DNAJC21

(DnaJ Heat Shock Protein Family (Hsp40) Member C21, Q5F1R6) is involved in ribosomal biogenesis and linked to mitochondrial function and stress responses. Its disruption impairs protein synthesis and affects tumor biology. Downregulation by VMG may impair ribosomal function and increase cellular stress, thereby hindering cancer cell survival.

### RNA Metabolism and Ribosome Biogenesis

Alterations in RNA processing and protein synthesis pathways are also targeted.

#### VIRMA

(Vir-like m6A methyltransferase associated, Q69YN4) is a component of the m6A RNA methyltransferase complex, which regulates RNA methylation and profoundly influences gene expression in cancer. Overexpression of VIRMA is associated with tumor growth and poorer prognosis ^[84]^. VMG-mediated downregulation of VIRMA can potentially alter the epitranscriptomic landscape of PDAC cells, disrupting oncogenic gene expression programs.

#### UTP4

(U3 small nucleolar RNA-associated protein 4 homolog, Q969X6) is a ribosome biogenesis factor crucial for the maturation of 18S rRNA and the assembly of the small ribosomal subunit. Dysregulated ribosome biogenesis is a hallmark of cancer, as it impacts protein synthesis rates and cellular proliferation ^[85–89]^. The suppression of UTP4 by VMG would limit the translational capacity of cancer cells, thereby impairing their growth and survival.

### Intracellular Trafficking and Organelle Homeostasis

VMG also impacts the complex network of intracellular membrane dynamics.

#### TRAPPC8

(Trafficking protein particle complex subunit 8, Q9Y2L5) is a component of the TRAPP complex, essential for ER-to-Golgi vesicular trafficking and vital for Golgi integrity, autophagy, and susceptibility to certain toxins. In various cancers, including colorectal carcinoma, TRAPPC8 modulates the trafficking of key proteins like PD-L1, influencing tumor progression and immune evasion. VMG-induced downregulation of TRAPPC8 likely disrupts intracellular transport and autophagic flux, pathways critical for tumor cell survival and adaptation, thereby contributing to tumor cell death ^[90–94]^.

#### ATL3

(Atlastin GTPase 3, Q6DD88) is an ER-resident GTPase involved in regulating ER membrane tethering and reticulophagy. Mutations and altered expression of ATL3 contribute to ER stress-related pathways and tumor resilience. VMG’s downregulation of ATL3 may induce ER stress, compromising cellular homeostasis and leading to cancer cell death ^[95]^.

#### ATP2C1

(Secretory Pathway Ca2+/Mn2+-ATPase 1, P98194) encodes a calcium/manganese pump located in the Golgi apparatus, crucial for maintaining intracellular calcium homeostasis. Abnormal ATP2C1 activity is implicated in various cancers, and its dysregulation can impact cell adhesion, ER stress response, and apoptosis. VMG-mediated reduction of ATP2C1 expression suggests a disruption of calcium signaling, potentially altering cancer cell viability and therapeutic sensitivity^[96–99]^.

### Cytoskeletal Regulation and Cell Motility

VMG impacts proteins governing cell structure and movement, which are essential for metastasis.

#### FAM83D

(Family with Sequence Similarity 83, Member D, Q9H4H8) promotes cell proliferation, invasion, and metastasis, and is associated with activating oncogenic pathways like PI3K/AKT. High expression of FAM83D correlates with advanced tumor stage and poor patient prognosis in PDAC and other cancers. VMG-induced downregulation of FAM83D suggests an inhibition of cancer cell aggressiveness by disrupting these proliferation and metastatic pathways^[100,101,101]^.

#### DIAPH3

(Diaphanous-Related Formin 3, Q9NSV4) is a formin family member that regulates the actin cytoskeleton, thereby influencing cell morphology, migration, and invasion. While its deficiency can enhance cell motility in some contexts, DIAPH3 has also been identified as an oncogene in certain cancers, promoting progression by activating antioxidant effects or inactivating mTOR signaling. VMG-mediated downregulation of DIAPH3 could therefore hinder tumor cell migration and invasion by disrupting actin dynamics and associated signaling pathways, leading to favorable outcomes in PDAC^[102–104]^.

#### ZDHHC5

(Zinc Finger DHHC-Type Containing 5, Q9C0B5) is a palmitoyltransferase that modifies various intracellular proteins, influencing their localization, stability, and function, including oncogenic RAS. Overexpression of ZDHHC5 in p53-mutant gliomas drives malignant development, and its depletion has been shown to inhibit cell growth in non-small cell lung cancer. The downregulation of ZDHHC5 by VMG may thus disrupt key oncogenic signaling pathways by altering protein palmitoylation, thereby impeding cancer cell growth and survival^[105–107]^.

#### SLC35E1

(Solute carrier family 35 member E1, Q96K37), though less comprehensively characterized, is a solute carrier that has been implicated in keratinocyte proliferation through its role in zinc homeostasis. Its altered expression in various cancers, such as endometrial cancer, suggests its involvement in cellular proliferation pathways. VMG-induced downregulation of SLC35E1 may correlate with a decreased proliferative capacity in PDAC cells^[108]^.

#### TTC14

(Tetratricopeptide repeat protein 14, Q96N46) contains tetratricopeptide repeat domains that mediate protein-protein interactions. While research on its direct role in cancer is still emerging, TTC14 mRNA is upregulated in lung adenocarcinoma and has been identified as a target of antibodies against tumor antigens. Its downregulation by VMG could potentially interfere with oncogenic protein interactions, modulate immune recognition, or affect cell cycle progression and apoptosis, thereby contributing to tumor suppression^[109]^.

### Upregulated Proteins: Facilitating Anti-Tumor Responses

Conversely, VMG infection also leads to the upregulation of specific proteins associated with a favorable prognosis in PDAC, indicating potential mechanisms through which the virus promotes tumor suppression or a less aggressive phenotype.

### Endosomal Sorting and Immune Regulation

VMG infection promotes the expression of a key protein involved in intracellular trafficking and immune modulation.

#### TOM1L2

(TOM1-like protein 2, Q6ZVM7) is an adaptor protein crucial for endosomal sorting and intracellular trafficking, interacting with Tollip, clathrin, and ubiquitin. It plays a role in the retrieval and degradation of ubiquitinated cargo, including G protein-coupled receptors, from cilia, which is essential for proper cellular signaling. Upregulation of TOM1L2 in VMG-infected cells suggests enhanced regulation of receptor internalization and turnover, potentially attenuating mitogenic pathways that drive PDAC progression. Studies in hypomorphic mouse models of Tom1l2 show increased tumor incidence and abnormal immunological responses, implying a tumor-suppressive role for TOM1L2, possibly through supporting immune functions or negatively regulating aberrant cell proliferation signals. This positions TOM1L2 as a mediator through which VMG may modulate oncogenic signals and enhance immune surveillance within the tumor microenvironment^[52,110–112]^.

#### Tumor Suppressor Pathway Modulation

Other upregulated proteins contribute to chromatin remodeling and stress responses.

#### MAGED2

(Melanoma Antigen Family D2, Q9UNF1) interacts with and negatively regulates the p53 tumor suppressor protein, influencing cell cycle control and apoptotic responses. While its role in suppressing TRAIL-R2 expression can confer resistance to apoptosis in melanoma, its upregulation in the context of VMG-treated PDAC cells might reflect a different outcome, potentially enhancing p53 pathway activity, promoting tumor suppression, and sensitizing cancer cells to stress-induced cell death. This dual nature suggests a complex role in cellular stress adaptation and anti-tumor responses^[113–118]^.

#### MBD3

(Methyl-CpG-binding Domain Protein 3, O95983) is a core component of the NuRD (Nucleosome Remodeling and Deacetylase) chromatin remodeling complex, which controls transcriptional regulation and maintains an active epigenetic state. While MBD3 has been implicated in various aspects of cancer, including promoting metastatic outgrowth by stabilizing MYC in PDAC, its upregulation in VMG-treated cells is associated with a favorable prognosis, suggesting a context-dependent role. This could involve beneficial epigenetic regulation, a shift towards tumor-suppressive pathways, or modulation of transcriptional programs that favor immune responses, proliferation control, and differentiation^[119–121]^.

### Major Affected Cellular Pathways

The comprehensive analysis of these dysregulated proteins reveals a convergence on several critical cellular pathways that are fundamentally implicated in PDAC pathogenesis and progression^[122,123]^. VMG’s ability to modulate these pathways highlights the broad therapeutic potential of this oncolytic approach.

#### Mitochondrial Bioenergetics and Metabolic Reprogramming:

The downregulation of MRPL3, TMEM70, and SQOR collectively indicates a disruption of mitochondrial function and energy metabolism^[65,68,69,124]^. PDAC cells often reprogram their metabolism to fuel rapid growth, making these pathways attractive therapeutic targets for VMG^[65,125–127]^.

#### DNA Replication, Repair, and Cell Cycle Control:

RRM2, NDC1, MIS18A, and LRIF1 are directly involved in DNA synthesis, cell division, and chromosome segregation. Dysregulation of these processes is a hallmark of cancer, and their modulation by VMG can induce cell cycle arrest, genomic instability, or apoptosis^[58,127–129]^.

#### Ubiquitin-Mediated Proteostasis and Epigenetic Regulation:

The modulation of proteins like ATAD2, MORF4L1, VIRMA, MAGED2, and MBD3 indicates complex alterations at the post-translational and epigenetic levels. This suggests VMG’s ability to alter oncogenic chromatin dynamics, protein stability, and gene expression programs that suppress tumor growth or enhance therapeutic sensitivity^[116,117,130–132]^.

#### RNA Processing and Ribosome Biogenesis:

UTP4 and CEBPZ are critical for ribosomal RNA (rRNA) processing and ribosome assembly. VMG’s impact on these proteins likely leads to impaired protein synthesis, which is a key vulnerability for rapidly proliferating cancer cells^[86,87,89]^.

#### Intracellular Trafficking and Organelle Homeostasis:

The upregulation of TOM1L2 suggests enhanced endosomal sorting and receptor turnover. Conversely, the downregulation of TRAPPC8 and ATL3 indicates disrupted ER-to-Golgi trafficking, which can lead to impaired autophagy, compromised protein secretion, and increased cellular stress —critical factors for tumor cell survival and adaptation^[90,93,94,111,112]^.

#### Calcium Homeostasis:

The downregulation of ATP2C1 suggests interference with cellular calcium signaling, a fundamental process regulating cell proliferation, differentiation, and apoptosis, which is often dysregulated in cancer^[95,96]^.

#### Cytoskeletal Dynamics and Cellular Motility:

Downregulation of FAM83D, DIAPH3, and ZDHHC5 indicates an impact on cytoskeletal remodeling, which may reduce PDAC cell migration, invasion, and metastatic potential^[100–102,133]^.

### VMG’s Mechanism and Translational Relevance

VMG, with its modified glycoprotein, demonstrates a multifaceted oncolytic mechanism in PDAC cells. The virus selectively replicates within tumor cells, leading to their lysis, and the released progeny viruses can then infect neighboring tumor cells. This direct oncolytic effect is complemented by a profound proteomic reprogramming of the host cell, which correlates with prognostic indicators in PDAC.

The downregulation of proteins associated with unfavorable prognosis—including those involved in mitochondrial function (ATAD2, TMEM70, MRPL3, SQOR), DNA synthesis and repair (RRM2), RNA modification (VIRMA), ribosome biogenesis (UTP4, DNAJC21, CEBPZ), intracellular trafficking (TRAPPC8, ATL3), cell cycle control (MIS18A, LRIF1, NDC1), cytoskeletal dynamics (FAM83D, DIAPH3), and palmitoylation (ZDHHC5)—collectively indicates that VMG effectively disrupts multiple fundamental processes driving PDAC progression, survival, and intrinsic chemoresistance.

Conversely, the upregulation of proteins linked to favorable prognosis, such as TOM1L2 (involved in endosomal sorting and immune regulation), MAGED2 (modulating p53 pathways), and MBD3 (a chromatin remodeler), suggests a potentiation of anti-tumor responses and a shift towards a less aggressive phenotype. This dual action of VMG—direct oncolysis and immunomodulation—is critical for its therapeutic promise.

From a translational perspective, these findings underscore VMG’s relevance in precision oncology. The infection-induced proteomic signature correlates with improved prognostic markers, suggesting that VMG could serve both as a direct therapeutic agent and as a modulator of the tumor microenvironment, potentially enhancing the efficacy of other therapies. Monitoring the expression levels of these specific proteins could provide invaluable pharmacodynamic markers to track treatment response, predict patient outcomes, and guide personalized therapeutic regimens in PDAC. VMG’s ability to downregulate proteins associated with tumor aggressiveness while enhancing anti-tumor immune-related proteins suggests a promising avenue for integration with existing or novel therapeutic modalities, including immunotherapies. These novel findings, together with existing knowledge of resistance mechanisms in PDAC, offer valuable guidance for bioengineering VMG with additional transgenes to enhance its therapeutic efficacy.

### Limitations and Future Directions

Despite the compelling findings, proteomic analyses in virotherapy studies face inherent limitations. Technical challenges include the dynamic range of protein abundance, difficulty in detecting low-abundance proteins, and accurately capturing transient post-translational modifications and protein-protein interactions. The updated protein dataset, with newly incorporated proteins such as ATAD2, TMEM70, LRIF1, and TRAPPC8, further emphasizes the intricate and multifaceted nature of VMG’s impact on PDAC cells. This expanded complexity necessitates sophisticated integrative analyses to distinguish direct viral effects from secondary cellular responses and to determine which protein changes are most critical for therapeutic efficacy and prognostic value.

Biologically, the substantial heterogeneity of PDAC tumors and the dynamic interplay between tumor cells and the immune microenvironment further complicate data interpretation ^[123]^. While transcriptomic correlation strengthens the findings, protein levels do not always directly correlate with mRNA expression, underscoring the need for multi-omics integration.

From a translational perspective, the inclusion of newly identified prognostic proteins with diverse biological functions creates significant opportunities for combinatorial therapeutic strategies but also demands careful mechanistic elucidation to anticipate and mitigate unforeseen antagonistic interactions. For example, combining VMG therapy with metabolic drugs targeting mitochondrial ATP synthase biogenesis (due to TMEM70 downregulation) or epigenetic drugs targeting ATAD2 and MORF4L1 could enhance outcomes, but this requires rigorous preclinical validation in physiologically relevant models.

Future research should focus on several key areas to overcome these limitations and fully realize the clinical potential of VMG:

#### In Vivo Validation:

Extensive studies utilizing patient-derived xenograft (PDX) and organoid (PDO) models, which more faithfully recapitulate human PDAC heterogeneity and microenvironment, are crucial to confirm the therapeutic efficacy of VMG and the prognostic value of these proteins in a complex physiological setting.

#### Mechanistic Elucidation:

Further investigations are needed to precisely define how VMG infection leads to the observed proteomic changes, including specific viral-host protein interactions, the intricate molecular mechanisms governing the favorable shifts, and the temporal dynamics of these changes.

#### Combinatorial Strategies:

Given the multifactorial nature of PDAC resistance, exploring combinatorial therapies integrating VMG with other targeted agents (e.g., RRM2 inhibitors, epigenetic modulators) or immunotherapies (e.g., PD-1/PD-L1 blockade) is a critical next step to enhance VMG’s oncolytic potential and simultaneously disrupt compensatory pathways. Moreover, VMG can be bioengineered to express transgenes that can improve its therapeutic efficacy.

#### Longitudinal Studies:

Implementing longitudinal monitoring of these protein biomarkers in preclinical models and eventually in human clinical trials will be essential to track disease progression, assess therapeutic response, and predict patient outcomes dynamically.

#### Advanced Proteomics and Multi-omics Integration:

Adopting advanced proteomics technologies with improved sensitivity, quantitative accuracy, and capacity to resolve dynamic proteoforms, coupled with the integration of transcriptomic, metabolomic, and immune profiling, will provide a more holistic understanding of VMG’s impact. This will also facilitate the development of predictive models for personalized VMG-based therapeutic regimens.

In summary, VMG elicits a comprehensive reprogramming of PDAC proteomic landscapes, suppressing key oncogenic factors while bolstering tumor suppressive and immune-related proteins, substantiating its potential clinical utility in pancreatic cancer treatment.

## Supplementary Material

Supplementary Files

This is a list of supplementary files associated with this preprint. Click to download.

- TableS1.pdf
- TableS2GOMFBPCC.xlsx

## Figures and Tables

**Figure 1.**
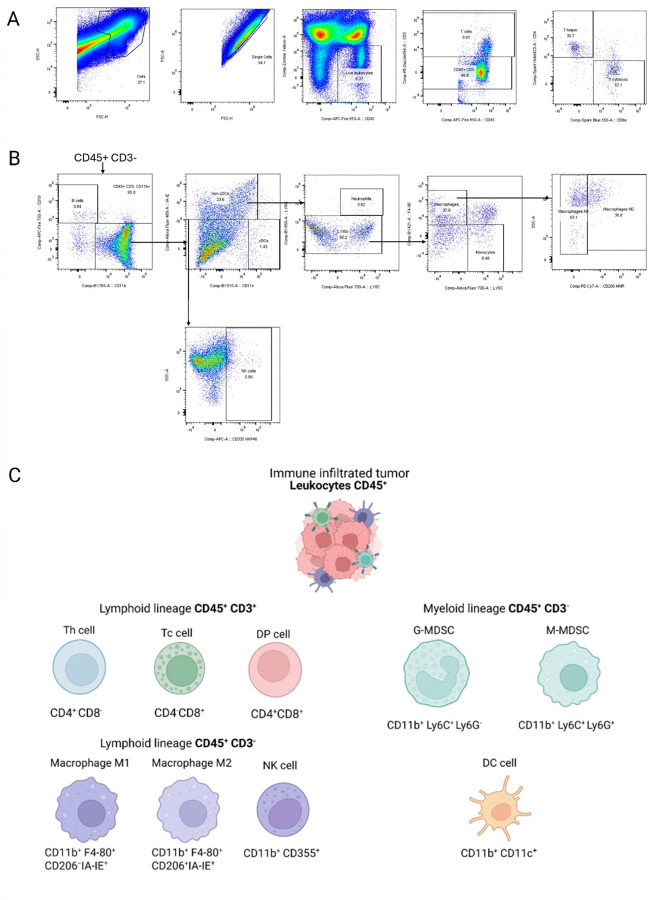
Representative gating schemes and immune population frequencies are shown in Figures 1A and 1B, with immune marker expression summarized in Figure 1C.

**Figure 5.**
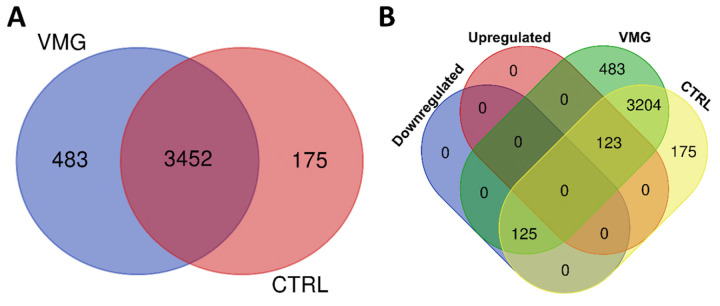
Comparison of detected proteins in VMG-treated vs. untreated control MiaPaCa2 cells. A) Venn diagram of detected proteins in the VMG-treated and control groups. 3,935 and 3,627 proteins were detected in the VMG-treated and control groups, respectively, with 3,452 of these proteins detected in both groups. B) Venn diagram illustrating the overlap of upregulated and downregulated proteins in VMG-treated and control groups.

**Figure 6.**
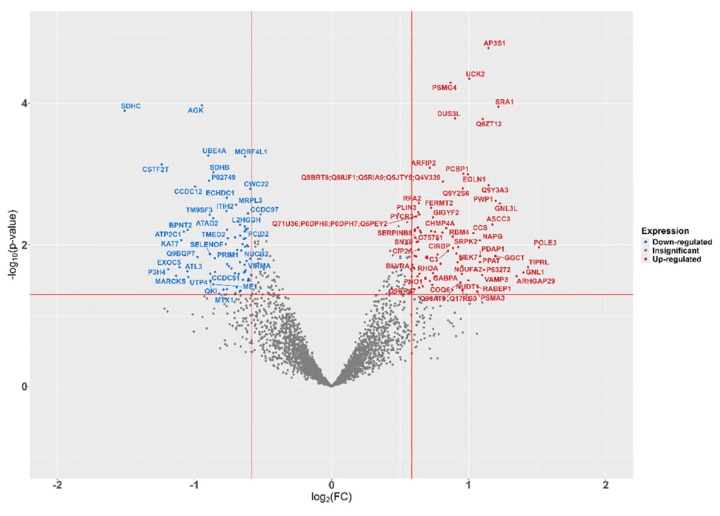
Volcano plot depicting commonly detected proteins significantly altered by VMG treatment.

**Table 1.** Functional enrichment of significantly altered proteins following VMG treatment. The top 5 Gene Ontology (GO) terms in each ontology (molecular function, biological process, and cellular component) are presented in each column.

Molecular Function (MF)			Biological Process (BP)			Cellular Component (CC)		
GO term	#	p-value	GO term	#	p-value	GO term	#	p-value
GO:000372 RNA binding	49	< 0.001	GO:0017148 negative regulation of translation	10	< 0.001	GO:000582 cytosol	125	< 0.001
GO:0005515 protein binding	225	< 0.001	GO:1901911 adenosine 5’-(hexahydrogen pentaphosphate) catabolic process	4	< 0.001	GO:0005654 nucleoplasm	100	< 0.001
GO:0016787 hydrolase activity	17	< 0.001	GO:1990705 cholangiocyte proliferation	4	< 0.001	GO:000563 nucleus	122	< 0.001
GO:0034431 bis(5’-adenosyl)-hexaphosphatase activity	4	< 0.001	GO:2001204 regulation of osteoclast development	4	< 0.001	GO:000573 cytoplasm	113	< 0.001
GO:0008486~dip hosphoinositol-polyphosphate diphosphatase activity	4	< 0.001	GO:0071543~di phosphoinositol polyphosphate metabolic process	4	< 0.001	GO:000579 Golgi apparatus	31	< 0.001

**Table 2.** Enriched KEGG pathways of significantly dysregulated proteins in VMG-treated samples compared to control samples (p-value < 0.05).

KEGG ID	Description	Category	Subcategory	p-value	Entrez ID	#
hsa03420	Nucleotide excision repair	Genetic Information Processing	Replication and repair	< 0.001	RAD23A, CCNH, RPA2, POLR2G, POLR2L, POLE3	6
hsa03030	DNA replication	Genetic Information Processing	Replication and repair	< 0.001	PRIM1, RPA2, RNASEH2A, POLE3	4
hsa00640	Propanoate metabolism	Metabolism	Carbohydrate metabolism	0.0041	ACOX1, HIBCH, ECHDC1	3
hsa00920	Sulfur metabolism	Metabolism	Energy metabolism	0.0044	BPNT2, SQOR	2
hsa01200	Carbon metabolism	Metabolism	Global and overview maps	0.0069	ACOX1, HIBCH, SDHB, ME1, SDHC	5
hsa04146	Peroxisome	Cellular Processes	Transport and catabolism	0.0103	ACOX1, NUDT19, HACL1, DECR2	4
hsa00190	Oxidative phosphorylation	Metabolism	Energy metabolism	0.0136	SDHB, ATP6V1C1, NDUFA2, NDUFB9, SDHC	5
hsa03460	Fanconi anemia pathway	Genetic Information Processing	Replication and repair	0.0187	FANCI, RPA2, BLM	3
hsa04110	Cell cycle	Cellular Processes	Cell growth and death	0.0231	ANAPC5, CCNH, CDK4, RB1, E2F4	5
hsa04145	Phagosome	Cellular Processes	Transport and catabolism	0.0236	C3, ATP6V1C1, STX7, RAB5B, VAMP3	5
hsa03082	ATP-dependent chromatin remodeling	Genetic Information Processing	Chromosome	0.0321	BAZ2A, MBD3, MORF4L1, POLE3	4
hsa00020	Citrate cycle (TCA cycle)	Metabolism	Carbohydrate metabolism	0.0376	SDHB, SDHC	2
hsa00410	beta-Alanine metabolism	Metabolism	Metabolism of other amino acids	0.0400	ACOX1, HIBCH	2
hsa04130	SNARE interactions in vesicular transport	Genetic Information Processing	Folding, sorting and degradation	0.0448	STX7, VAMP3	2
hsa03020	RNA polymerase	Genetic Information Processing	Transcription	0.0473	POLR2G, POLR2L	2

**Table 3.** IPA canonical pathways of significantly dysregulated proteins in VMG-treated samples compared with control samples. The top 20 canonical pathways are ranked by p-value, with columns indicating the p-value for pathway enrichment, the ratio of identified proteins to total pathway molecules, predicted activation z-score, and the dysregulated molecules in each pathway.

Ingenuity Canonical Pathways	p-value	z-score	Ratio	Molecules
Mitotic G1 phase and G1/S transition	< 0.001	1.414	0.0682	CCNH, CDK4, E2F4, PRIM1, PSMA3, PSMB10, RB1, RRM2, TFDP1
RNA Polymerase II Transcription	< 0.001	0.333	0.06	CCNH, CDC40, CSTF2T, ELOB, GTF2E2, POLR2G, POLR2L, SRSF5, SUPT4H1
NER (Nucleotide Excision Repair, Enhanced Pathway)	< 0.001	0.816	0.0769	CCNH, COPS8, POLE3, POLR2G, POLR2L, PRIM1, RPA2
S Phase	< 0.001	0.378	0.07	CCNH, CDK4, E2F4, PSMA3, PSMB10, RB1, TFDP1
TP53 Regulates Transcription of DNA Repair Genes	< 0.001	0.816	0.0857	CCNH, ELOB, FANCI, POLR2G, POLR2L, SUPT4H1
Huntington’s Disease Signaling	< 0.001	NA	0.0383	ARFIP2, GNG5, HTT, NAPG, POLR2G, POLR2L, PSMA3, PSMB10, SDHB, SDHC, VAMP3
Nucleotide Excision Repair	< 0.001	1.89	0.0642	CCNH, COPS8, POLE3, POLR2G, POLR2L, RAD23A, RPA2
Processing of Capped Intron-Containing Pre-mRNA	< 0.001	−0.905	0.0382	CCDC12, CDC40, CSTF2T, CWC22, NDC1, PCBP1, POLR2G, POLR2L, PRCC, RBM7, SRSF5
Estrogen-mediated S-phase Entry	< 0.001	NA	0.154	CDK4, E2F4, RB1, TFDP1
RNA polymerase II transcribes snRNA genes	< 0.001	−0.816	0.0723	GTF2E2, INTS10, INTS14, POLR2G, POLR2L, SUPT4H1
Synthesis of DNA	< 0.001	1.342	0.0574	ANAPC5, GINS4, POLE3, PRIM1, PSMA3, PSMB10, RPA2
Peroxisomal lipid metabolism	< 0.001	−2	0.138	ACOX1, DECR2, HACL1, NUDT19
DYRK1A Signaling Pathway	< 0.001	1.414	0.0465	CDK4, E2F4, ELOB, NOTCH2, POLR2G, POLR2L, RB1, TFDP1
Class I MHC-mediated antigen processing and presentation	< 0.001	0.905	0.0315	ANAPC5, ELOB, ERAP1, GAN, LNPEP, NEDD4, PSMA3, PSMB10, SEC24B, TRIM32, UBE4A, VAMP3
Protein Ubiquitination Pathway	< 0.001	0.333	0.036	ANAPC5, DNAJC21, ELOB, NEDD4, PSMA3, PSMB10, UBE4A, USP15, USP28, USP4
Nucleotide Excision Repair Pathway	< 0.001	NA	0.108	CCNH, POLR2G, POLR2L, RPA2
Maturation of TCA enzymes and regulation of the TCA cycle	< 0.001	NA	0.15	ISCA1, SDHB, SDHC
Cyclins and Cell Cycle Regulation	< 0.001	1.342	0.0581	CCNH, CDK4, E2F4, RB1, TFDP1
Assembly of RNA Polymerase II Complex	0.001023	0	0.0784	CCNH, GTF2E2, POLR2G, POLR2L
Telomere Maintenance	0.001122	−0.447	0.0562	BLM, POLR2G, POLR2L, PRIM1, RPA2

**Table 4.** VMG-Dysregulated Proteins Overlapping with TCGA Pancreatic Adenocarcinoma Prognostic Markers (Downregulated-Unfavorable and Upregulated-Favorable). The results shown here are based upon data generated by the TCGA Research Network: https://www.cancer.gov/tcga. Bolded proteins are validated prognostic markers.

	Entry	Gene	Major Affected Pathways
**Downregulated Unfavorable**	P09001	MRPL3	Mitochondrial translation
	P31350	RRM2	G1/S Transition
	P98194	ATP2C1	Intracellular calcium homeostasis
	Q5F1R6	DNAJC21	Ribosome biogenesis
	Q69YN4	VIRMA	RNA processing
	Q6DD88	ATL3	Intracellular trafficking
	Q9BTX1	NDC1	Nuclear pore complex assembly
	Q9C0B5	ZDHHC5	Protein palmitoylation
	Q9H4H8	FAM83D	G1/S Transition
	Q9NSV4	DIAPH3	Mitosis
	Q9NYP9	MIS18A	Mitosis
	Q9Y6N5	SQOR	Oxidative phosphorylation
	Q6PL18	ATAD2	Epigenetic regulation
	Q03701	CEBPZ	Ribosome biogenesis
	Q5T3J3	LRIF1	Mitosis
	Q969X6	UTP4	Ribosome biogenesis
	Q96K37	SLC35E1	Transmembrane transport
	Q96N46	TTC14	Protein-protein interaction
	Q9BUB7	TMEM70	Oxidative phosphorylation
	Q9UBU8	MORF4L1	Transcriptional activation
	Q9Y2L5	TRAPPC8	Intracellular trafficking
**Upregulated Favorable**	O95983	MBD3	RNA pol II transcription
	Q6ZVM7	TOM1L2	Intracellular trafficking
	**Q9UNF1**	MAGED2	RNA pol II transcription

## Data Availability

The authors confirm that all data supporting the study’s findings are included within the article and its supplementary materials. Additionally, all data can be obtained from the corresponding author upon reasonable request.
